# Trends in Research on Traditional Chinese Health Exercises for Improving Cognitive Function: A Bibliometric Analysis of the Literature From 2001 to 2020

**DOI:** 10.3389/fpubh.2021.794836

**Published:** 2022-01-06

**Authors:** Wenlong Li, Linman Weng, Qiuping Xiang, Tonggang Fan

**Affiliations:** ^1^College of Chinese Wushu, Shanghai University of Sport, Shanghai, China; ^2^Department of Sport Rehabilitation, Shanghai University of Sport, Shanghai, China; ^3^Yueyang Hospital of Integrated Traditional Chinese and Western Medicine, Shanghai University of Traditional Chinese Medicine, Shanghai, China

**Keywords:** bibliometric analysis, cognitive function, Tai Chi, traditional Chinese health exercises, research trends

## Abstract

Although previous studies have investigated the ability of traditional Chinese health exercises (TCHEs) to improve cognitive function, few have utilized bibliometric analyses to address this topic. We aimed to investigate the current status of and developmental trends in this field from 2001 to 2020. We searched the Web of Science Core Collection (WoSCC) for all research publications on cognitive function in relation to TCHEs. CiteSpace V was used to analyze the number of papers, countries, institutions, journals, authors, and citations. We identified hotspots and trends in the field by drawing co-citation reference and co-occurrence keyword maps. From 2001 to 2020, 406 relevant articles were published in the WoSCC, with a gradual increase in the annual number of publications. The three countries/regions with the most publications were the Chinese mainland, the United States, and Canada. Six universities from China and four from the United States were identified as the top 10 institutions. Most research was conducted at universities. Evidence-based Complementary and Alternative Medicine was identified as the most productive journal. Together, these findings indicate that TCHEs have received increasing attention as a method for improving cognition.

## Introduction

Cognitive function is known to decrease with aging, manifesting as impairments in memory, language, judgement, and attention (1). When the decline exceeds the normal range for the patient's age group, the patient is considered to have mild cognitive impairment, which is likely to progress to dementia (2). Dementia is caused by damage to or destruction of neurons in brain areas involved in cognitive functions such as thinking, learning, and memory (3), and this disabling neurological disorder is common worldwide (4). According to global estimates, 35.6 million people lived with dementia in 2010, with numbers expected to rise to 65.7 million by 2030 and 115.4 million by 2050 (5). The annual economic burden of dementia is 1 trillion USD and is expected to double by 2030 (6). Moreover, as Alzheimer's disease is underdiagnosed among patients with dementia, the aforementioned numbers may be an underestimation (3).

Both cognitive decline and cognitive impairment affect an individual's social interactions and work performance (1, 7). As the condition worsens, activities such as walking and eating are affected, and the patient may even be confined to bed until death (3). At present, there is no cure for dementia, and current treatments can only delay the decline of cognitive ability to a certain extent (8). Furthermore, drug treatment induces strong side effects in many patients, which may lead to death (9). Studies have shown that non-drug treatments may have a more significant effect in patients with dementia than drug treatments (10). Therefore, non-drug therapies are of great significance for their potential to improve cognitive function.

The control of early risk factors is important for reducing the likelihood of dementia (11), and regular physical exercise has been shown to reduce the risk of both cognitive decline and dementia (12). One study demonstrated that aerobic training can increase hippocampal volume in patients with mild cognitive impairment, thereby improving cognitive function (13). Other studies have reported that performing aerobic exercise for no <20 min and at least three times a week for 3 months can trigger neurogenesis, thus promoting the optimization of brain plasticity, which is beneficial for cognitive function (14). Traditional Chinese health exercises (TCHEs) are performed at moderate aerobic intensity and have been considered safe in the context of medical treatment (15, 16). Moreover, TCHEs are based on traditional Chinese medicine; hence, they provide the benefits of both exercise and medical treatment (15). Compared with drug therapy and general exercise therapy, TCHEs are safer and less costly, making them suitable for most people to practice. Sungkarat et al. (17) suggested that the practice of TCHEs three times a week for 15 weeks can effectively improve cognitive function. Through a 1-year randomized controlled experiment, Jin et al. concluded that TCHEs not only prevent the decline of cognitive function but also improve cognitive function to a certain extent (18).

To date, numerous studies have investigated the ability of TCHEs to improve cognitive function. Such studies have reported promising results, including a decrease in the rate of cognitive decline in older adults (19), improvements in cognitive function in patients with mild cognitive impairment, and a decrease in the risk of dementia (20). However, few researchers have collected global data regarding the influence of TCHEs on cognitive function and the status of current research. Moreover, few studies have examined emerging trends from the perspective of visualization and bibliometric analysis. Such analyses are necessary for a more in-depth assessment of research in this field.

Bibliometric analysis is a quantitative method used to identify scientific activities and generate an organizational knowledge structure based on the processing of information related to journals, authors, institutions, etc. (21). These analyses can help to identify research hotspots and trends, in addition to providing a reference for future investigations (22, 23). The number of studies employing bibliometric methods to analyze research in specific fields tends to increase each year. Such methods have been applied in research areas such as low back pain, insomnia, and knee osteoarthritis (24–26). CiteSpace is a visualization tool for bibliometric and comparative analysis developed by Professor Chen Chaomei (27). CiteSpace can generate a co-occurrence network of countries, authors, institutions, keywords, cited authors, cited journals, and cited references (28). By presenting the data in the form of a knowledge map, this tool allows researchers to intuitively analyze developmental trends within the discipline and identify emerging frontiers (28).

In the present study, we conducted a bibliometric analysis of research regarding the effect of TCHEs on cognitive function based on studies published over the past 20 years. CiteSpace was used to assess the status of and developmental trends in this field, including collaborations and distributions among countries, institutions, and authors. We also performed a co-occurrence analysis of keywords and a citation analysis.

## Materials and Methods

### Data Collection and Search Strategy

All relevant articles published from 2001 to 2020 were retrieved from the Web of Science Core Collection (WoSCC) on 11 May 2021. The search strategy consisted of two parts. (1) Search keywords related to TCHEs, such as “Tai Chi,” “Baduanjin” or “Chinese traditional exercise,” were entered into the WoSCC. We used the Science Citation Index Expanded (SCI-E) and the Social Science Citation Index (SSCI) to search for original articles or reviews published in English. A total of 22,655 publications were retrieved. (2) Next, search keywords related to cognitive function, such as “cognition” or “cognitive function,” were entered into the WoSCC, and the SCI-E and SSCI were again used to identify original research articles or reviews published in English. We collected 156,167 records using this query. The first and second queries were combined, and 406 papers were obtained. The specific search strategies and results are listed in Table 1.

**Table 1:** Data sources.

**Content**			
Data sources	WoSCC (SCI-E, SSCI)		
Time span	2001–2020		
Languages	English		
Literature types	Article or review		
Search strategy	#1	22655	[TS = (tai-ji or “Tai Chi” or “Chi, Tai” or “Tai Ji Quan” or “Ji Quan, Tai” or “Quan, Tai Ji” or “Taiji” or “Taijiquan” or “T'ai Chi” or “Tai Chi Chuan” or qigong or “qi gong” or “chi gong” or “ch'i Kung” or “baduanjin” or “ba duan jin” or “wuqinxi” or “yijinjing” or “yi jinjing” or “yi jinjing” or “liuzijue” or “traditional exercise” or “traditional Chinese medicine” or “Chinese traditional exercise” or “traditional Chinese exercise” or “Chinese exercise” or “mind-body exercise”)]
	#2	156167	[TS = (cognitions or “cognitive function” or “cognitive functions” or “function, cognitive” or “functions, cognitive”)]
	#3	406	#2 AND #1

### Analysis Tool

CiteSpace V (5.6.R5) (29) was used to conduct a bibliometric analysis of 406 articles published from 2001 to 2020. CitiSpace V is a Java-based software program that builds knowledge network maps, including cooperation network maps, co-citation network maps, co-occurrence network maps, and dual-map overlays of journals (28, 30). The visualization network map includes nodes and links. The nodes represent countries, institutions, authors, and cited references, which we extracted from the Web of Science (WoS). A node with a large diameter indicates a high frequency of occurrence or citation of the country, institution, author, or cited reference. The links represent the cooperation, co-occurrence, and co-citation relationships between two nodes. A purple ring around nodes with high centrality values indicates that the node may be regarded as an important key point in the field (28, 31, 32).

The strategies for bibliometric analysis were as follows: (1) Occurrence burst or citation burst refers to a sharp increase in the frequency of occurrence or citation during a specific time span. The keywords or references with the strongest citation burst (i.e., the top-ranking keywords or references) were identified (burst detection) and considered to reflect relevant topics that gained considerable attention over a period of time, representing possible research hotspots or frontiers (33, 34). (2) Papers concerning similar topics or those with homogeneous characteristics were aggregated into clusters (clustering), which allowed us to capture different categories and examine their relationships in the field. (3) A prediction model [f(x) = ax^3^ + bx^2^ + cx +d] was then used to estimate future trends in research regarding TCHEs within the field of cognitive function. In this case, “x” represented the year of publication, and “f(x)” represented the number of papers published (27, 35).

## Results

### Analysis of Publication Outputs and Developmental Trends

**Figure 1:**
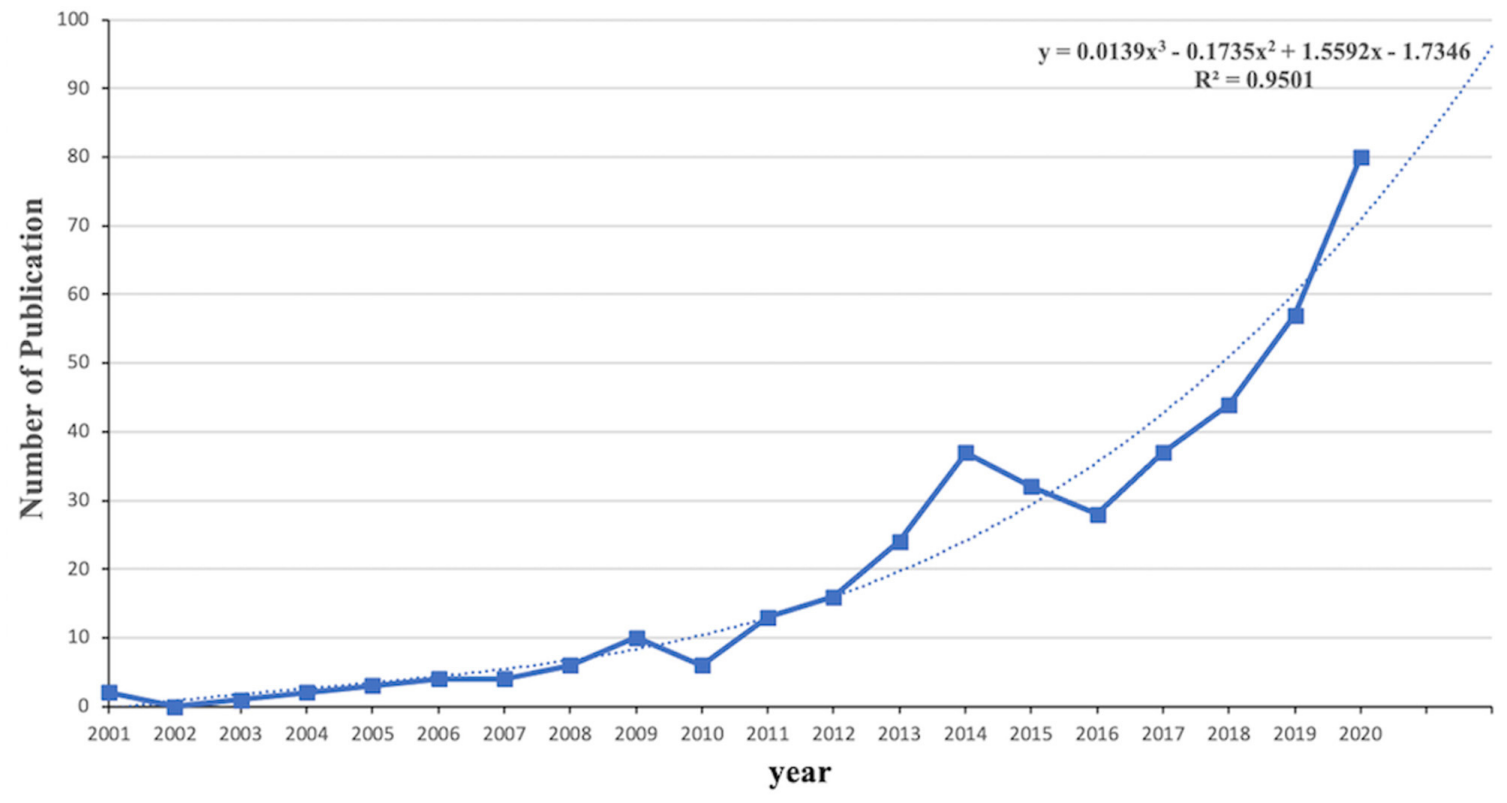
Distribution of publications and growth prediction from 2001 to 2020.

Our search retrieved a total of 406 records. During the 20-year publication period, the annual number of publications exhibited an upward trend, and the full period was divided into three stages based on fluctuations (Figure 1). The first stage occurred from 2001 to 2009, with an average of 3.5 papers published each year. Overall, the annual number of publications was lowest during this period and exhibited steady growth. In the second stage, which occurred from 2010 to 2014, the number of publications increased rapidly, with an average of 19.2 papers published per year. The third stage ranged from 2015 to 2020 and exhibited the fastest growth rate, with an average of 46.3 publications each year. More papers were produced during the third stage than during any other period. There were four periods in which we observed a slight decline in publication output: 2001–2002 (from 2 in 2001 to 0 in 2002), 2009–2010 (from 10 in 2009 to 6 in 2010), and 2014–2016 (from 37 in 2014 to 28 in 2016). The model fitting curve indicated a significant correlation between the publications per year and year (R^2^ = 0.9501). This model predicted that the number of papers in 2021 will be ~83. The total number of citations also exhibited a trend of gradual increase (from 0 in 2001 to 2,370 in 2020).

### Analysis of Countries/Regions and Institutions

**Figure 2:**
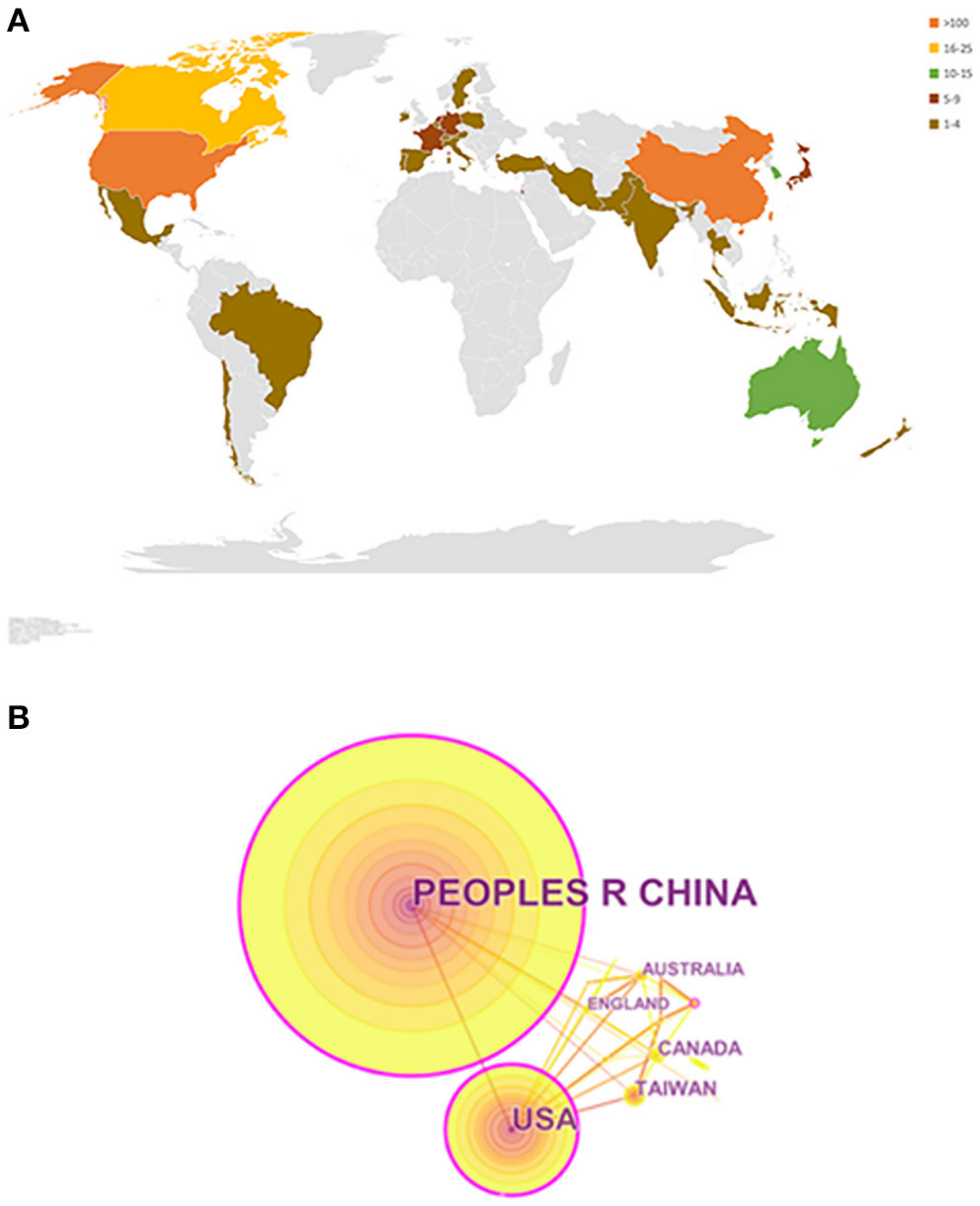
**(A)** World map of publications distributed in various countries/regions from 2001 to 2020. **(B)** Cooperation map of countries/regions from 2001 to 2020.

A total of 406 papers from 34 countries/regions were published from 2000 to 2020. The distribution of these 34 countries/regions is shown in Figure 2A, and the collaborations among them are shown in Figure 2B. Of the top 10 countries/regions with the largest number of papers, five were in Asia, two were in Europe, two were from the Americas, and one was from Oceania. The countries producing the most publications were the Chinese mainland (250 publications), the United States (104 publications), and Canada (22 publications), accounting for 92.6% of all papers. The United States (0.36), Chinese mainland (0.26), and Germany (0.15) were the top three countries/regions in terms of centrality (Table 2). These results indicate that the Chinese mainland and the United States were the key countries/regions in this field, having made remarkable progress and establishing close cooperative relationships.

**Table 2:** Top 10 countries/regions in terms of publications and centrality from 2001 to 2020.

**Ranking**	**Publications**	**Countries/Regions**	**Year**	**Ranking**	**Centrality**	**Countries/Regions**	**Year**
1	250	People R China	2007	1	0.36	USA	2005
2	104	USA	2005	2	0.26	People R China	2007
3	22	Canada	2013	3	0.15	Germany	2015
4	21	Taiwan	2016	4	0.08	Israel	2015
5	15	Australia	2015	5	0.06	Australia	2015
6	15	England	2015	6	0.05	Canada	2016
7	12	South Korea	2015	7	0.04	England	2015
8	8	Germany	2015	8	0.01	Taiwan	2013
9	8	Japan	2017	9	0.01	South Korea	2017
10	7	Israel	2017	10	0	Thailand	2017

**Figure 3:**
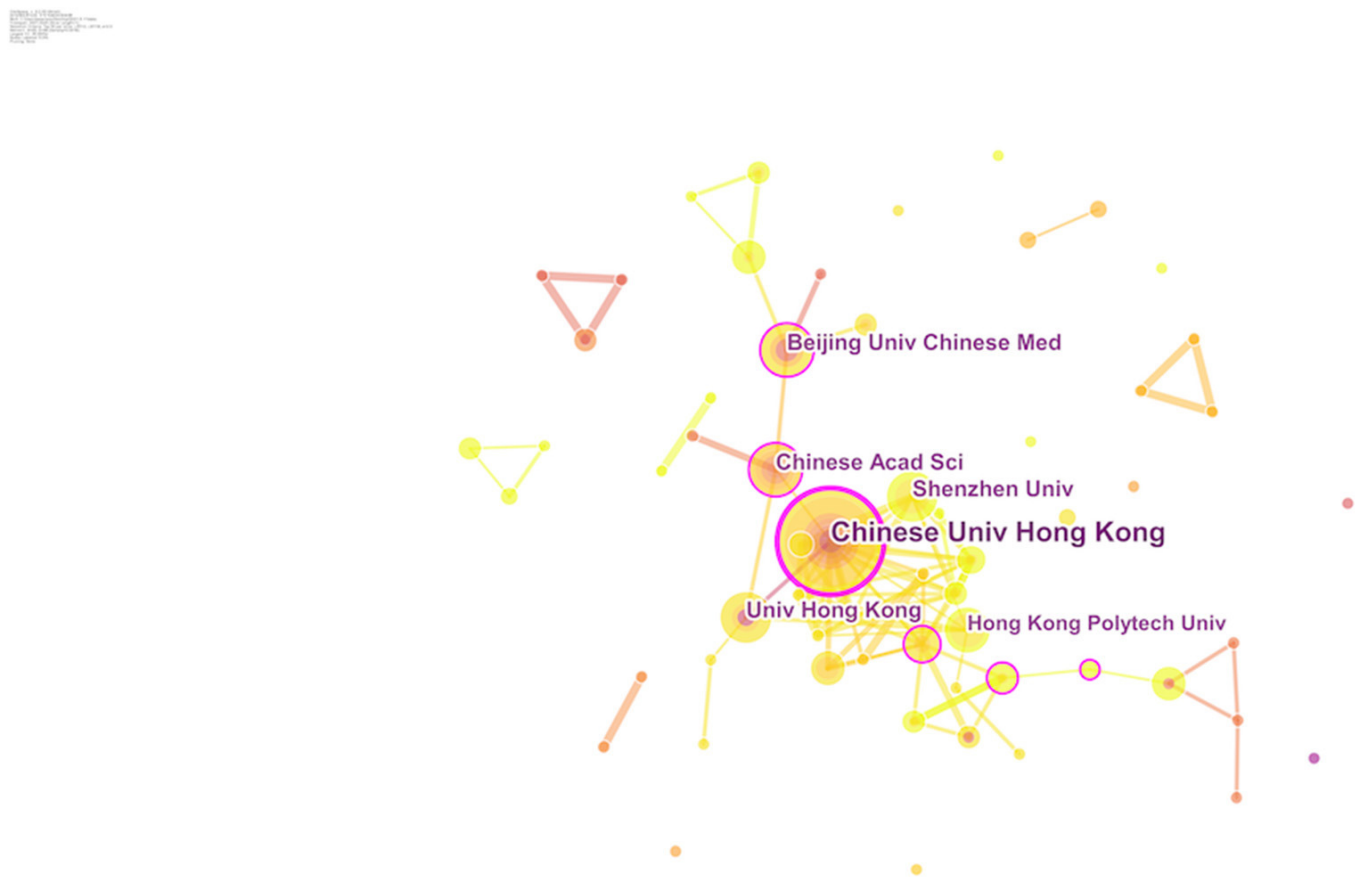
Cooperation map of institutions from 2001 to 2020.

A total of 662 institutions were involved in the development of the 406 research papers. Figure 3 shows the cooperation among institutions. Most of the top ten institutions were colleges and universities, six of which were in China, while the remaining four were in the United States. The Chinese University of Hong Kong (22 publications), Beijing University of Chinese Medicine (15 publications), Chinese Academy of Sciences (15 publications), Harvard University (15 publications), Hong Kong Polytechnic University (15 publications), and University of Hong Kong (15 publications) were the top six institutions with the largest number of publications. The Chinese University of Hong Kong (0.24) had the highest value of centrality, followed by Harvard Medical School (0.19) and the Chinese Academy of Sciences (0.17) (Table 3). In terms of publications and centrality, the Chinese University of Hong Kong, Harvard Medical School, and Chinese Academy of Sciences were the main research powers in this field.

**Table 3:** Top 10 institutions in terms of publications and centrality from 2001 to 2020.

**Ranking**	**Count**	**Institutions**	**Ranking**	**Centrality**	**Institutions**
1	22	Chinese University of Hong Kong	1	0.24	Chinese University of Hong Kong
2	15	Beijing University of Chinese Medicine	2	0.19	Harvard Medical School
3	15	Chinese Academy of Sciences	3	0.17	Chinese Academy of Sciences
4	15	Harvard University	4	0.13	Beijing University of Chinese Medicine
5	15	Hong Kong Polytechnic University	5	0.13	Shanghai Univ Med Hlth Sci
6	15	University of Hong Kong	6	0.11	Shanghai University of Traditional Chinese Medicine
7	11	Massachusetts General Hospital	7	0.08	Shanghai University of Sport
8	11	Nanjing University of Chinese Medicine	8	0.07	University of Hong Kong
9	11	University of Illinois System	9	0.06	Hong Kong Polytechnic University
10	10	University of California System	10	0.06	China Academy of Chinese Medical Sciences

### Analysis of Journals

The articles were published in more than 200 journals. The 10 most productive journals published 105 papers, accounting for 25.9% of the total number of papers identified (Table 4). Among these journals, *Evidence-based Complementary and Alternative Medicine* was the most productive journal (19 publications), followed by the *Journal of Ethnopharmacology* (17 publications) and *Neural Regeneration Research* (14 publications). *Frontiers in Aging Neuroscience* was the journal with the highest impact factor (IF, 2020 = 5.75), followed by the *Journal of the American Geriatrics Society* (IF, 2020 = 5.562) and *Neural Regeneration Research* (IF, 2020 = 5.135). Most journals were founded in the United States, and only one was founded in China. The IF of the top 10 journals mainly ranged between 1 and 5.

**Table 4:** Top 10 journals in terms of publications from 2001 to 2020.

**Ranking**	**Journal**	**Publications**	**Countries/Regions**	**IF (2020)**
1	Evidence Based Complementary and Alternative Medicine	19	England	2.629
2	Journal of Ethnopharmacology	17	Ireland	4.36
3	Neural Regeneration Research	14	China	5.135
4	Journal of Aging and Physical Activity	10	United States	1.961
5	Frontiers in Aging Neuroscience	9	United States	5.75
6	Medicine	8	United States	1.889
7	BMC Complementary and Alternative Medicine	7	England	3.659
8	Clinical Interventions in Aging	7	New Zealand	4.458
9	Frontiers in Human Neuroscience	7	Switzerland	3.169
10	Journal of The American Geriatrics Society	7	United States	5.562

**Figure 4:**
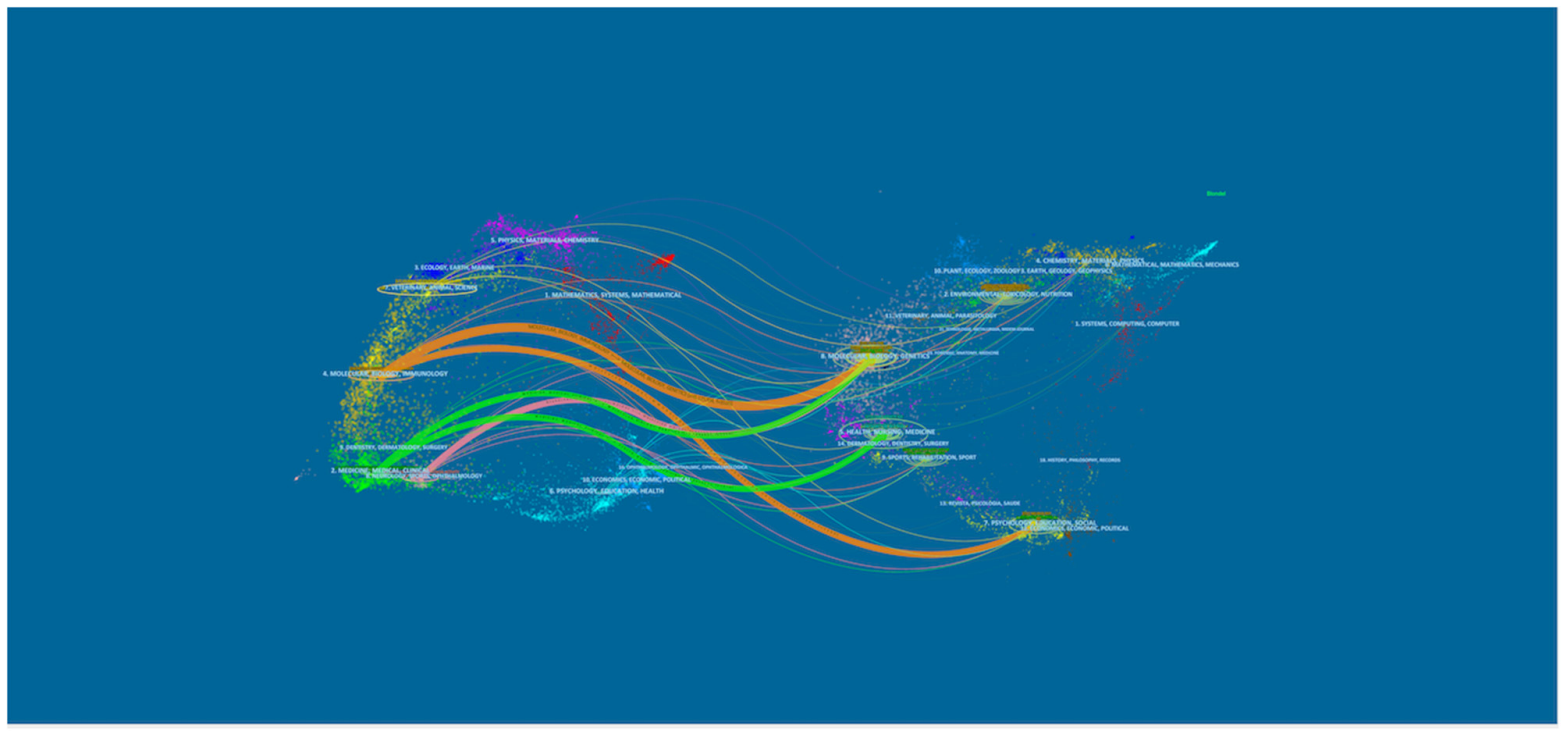
Dual-map overlay of academic journals from 2001 to 2020.

Figure 4 presents the dual-map overlay of academic journals and the citation paths between disciplines. The left labels of the map represent the disciplines of the citing journals, and the right labels represent the disciplines of the journals in which the cited papers were published. The majority of publications were published in journals related to molecular biology, immunology, medicine, neurology, sports, and ophthalmology. In addition, the majority of publications were cited in journals related to molecular biology, genetics, health, nursing, medicine, psychology, education, and social science.

### Analysis of References

**Figure 5:**
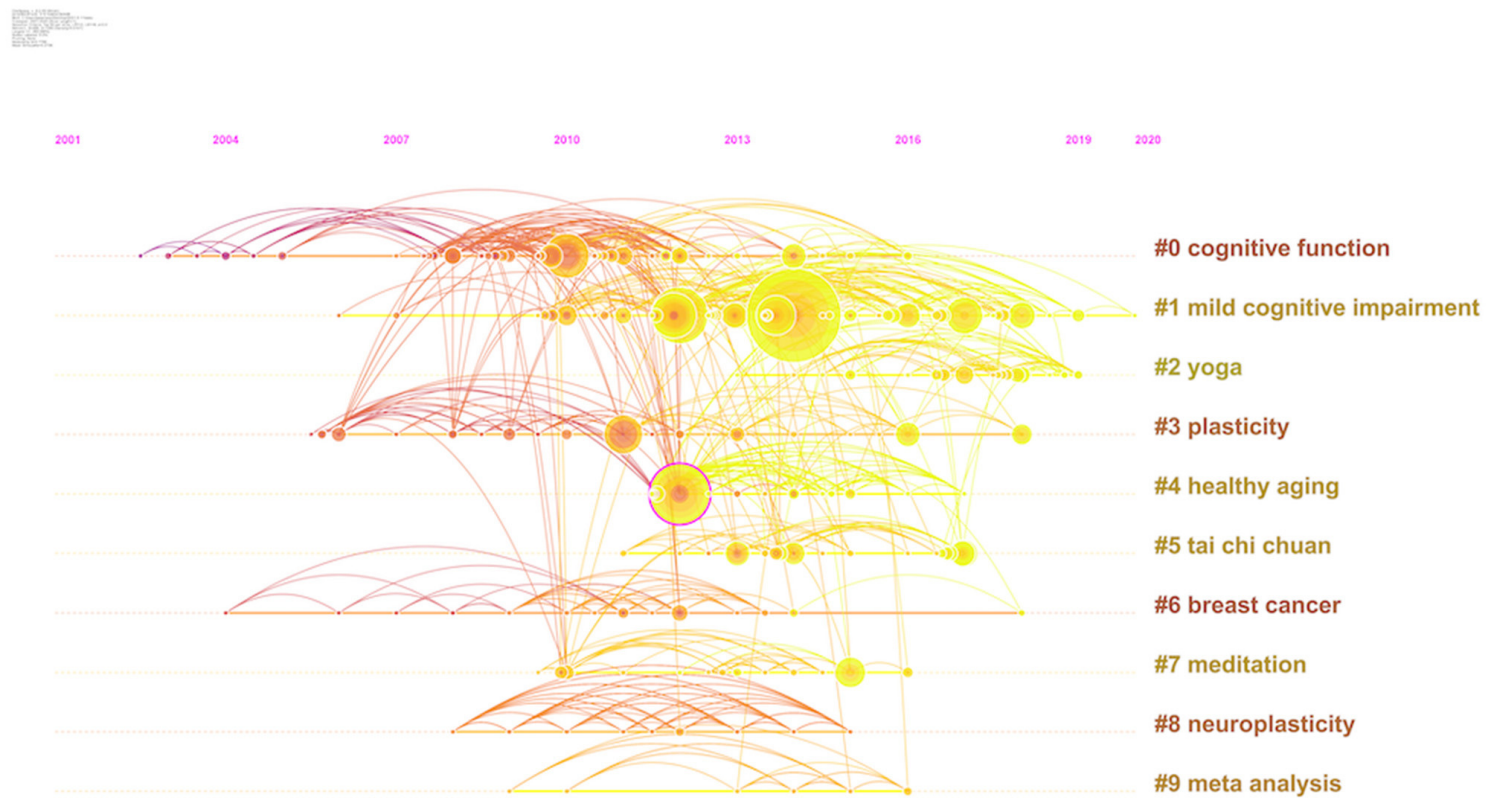
Timeline view of references from 2001 to 2020.

Reference analysis was used to assess basic knowledge and research frontiers in the field (36). The timeline view of the references shows nine clusters (Figure 5). Each cluster consisted of papers on similar topics. Cluster #0 (cognitive function) was the largest cluster, followed by cluster #1 (mild cognitive impairment). Many recently published papers were found in cluster #1, which also contained the references with the highest number of citations.

### Analysis of Keywords

**Figure 6:**
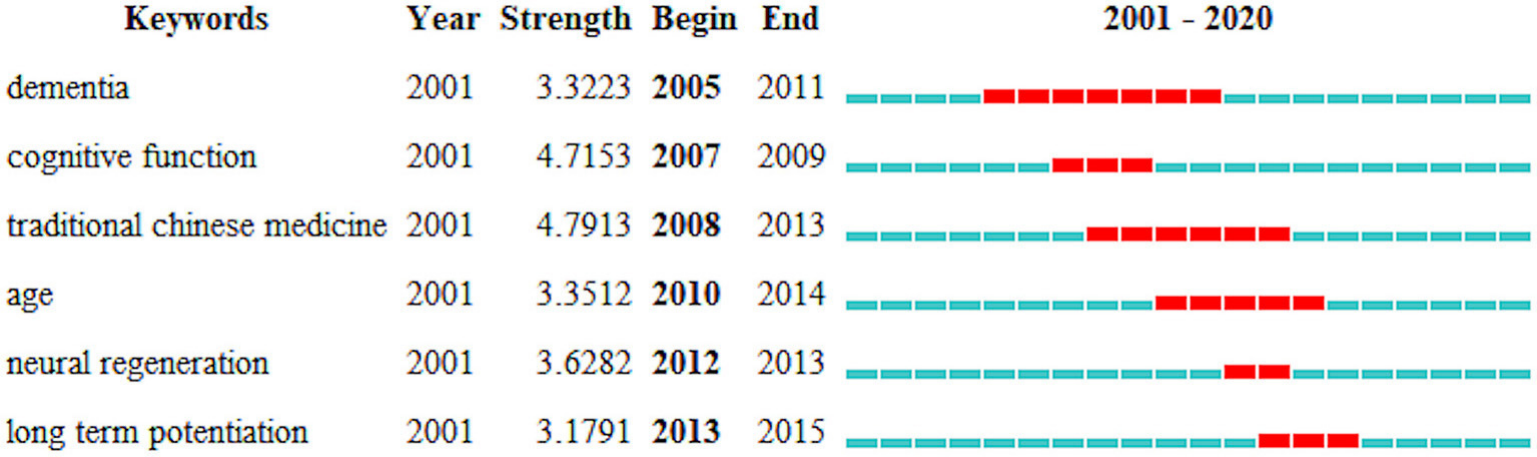
Top six keywords with the strongest citation bursts.

The keywords with the strongest citation burst and highest co-occurrence frequency may reflect hot topics in the research field. Figure 6 presents the six keywords most frequently cited in a certain period, which were “dementia,” “cognitive function,” “traditional Chinese medicine,” “age,” “neural regeneration,” and “long-term potentiation.” In terms of co-occurrence counts and centrality, “Alzheimer's disease,” “exercise,” “dementia,” “older adult,” “cognitive function,” and “quality of life” were the top keywords (Table 5).

**Table 5:** Top 10 keywords in terms of frequency and centrality from 2001 to 2020.

**Ranking**	**Count**	**Keywords**	**Ranking**	**Centrality**	**Keywords**
1	105	Alzheimer's disease	1	0.2	Traditional Chinese medicine
2	105	Tai chi	2	0.17	Cognitive impairment
3	75	Exercise	3	0.16	Alzheimer's disease
4	71	Cognition	4	0.16	Quality of life
5	69	Dementia	5	0.13	Older adult
6	69	Older adult	6	0.12	Brain
7	58	Cognitive function	7	0.12	Memory
8	57	Physical activity	8	0.11	Exercise
9	51	Impairment	9	0.1	Cognitive function
10	45	Quality of life	10	0.09	Dementia

## Discussion

In this study, we conducted a bibliometric analysis of research regarding the effects of TCHEs on cognitive function by examining articles published from 2001 to 2020. We observed a rapid increase in the number of publications and citations over the study period, which indicates that TCHEs have received increasing attention as a method for improving cognition. Overall, the current findings provide a reference for future research and highlight TCHEs as an option for managing cognitive decline.

Because of their origin in China, TCHEs have a greater influence in Asia than in other parts of the world. Of the top 10 countries/regions with the largest number of papers, five were in Asia, two were in Europe, two were in the Americas, and one was in Oceania. Among the top 10 institutions, six were in China, while the remaining four were in the United States. However, most journals were founded in the United States, and only one was founded in China, suggesting that Chinese journals should continue to improve their international competitiveness to attract more submissions. The IF of the top 10 journals mainly ranged between 1 and 5, which highlights the difficulty of publishing articles in journals with a higher IF.

### Cluster Analysis of References

The cluster analysis of references revealed that the data on this topic over the past 20 years are generally related to the following: (a) mild cognitive impairment, (b) yoga, (c) Tai Chi.

#### Mild Cognitive Impairment

Mild cognitive impairment refers to the intermediate stage marking the transition from normal age-related cognitive decline to dementia. In this stage, the damage is not severe and does not affect independent life (37). People who experience mild cognitive impairment are more likely to develop dementia than people with normal cognition (38). However, interventions and treatments are most effective when the degree of cognitive impairment is still mild. As many as 22% of patients with mild cognitive impairment gradually return to normal levels of cognitive function through early intervention (39). The guidelines for the diagnosis of dementia point out that adherence to aerobic exercise can improve cognitive function in patients with mild cognitive impairment and reduce the possibility of dementia. Medium-strength evidence also suggests that TCHEs can promote improvements in cognitive function in patients with mild cognitive impairment (40).

#### Yoga

Similar to TCHEs, yoga is a form of mind-body exercise (41) characterized by slow physical movement, full-body stretching and relaxation, breathing techniques, and mental concentration (42). Therefore, some studies have compared the effects of yoga and TCHEs on cognitive function (43). Research has shown that yoga and meditation-based lifestyle interventions enhance neuroplasticity and thereby improve cognitive function (44).

#### Tai Chi

Tai Chi is a representative type of TCHE that is often used to promote cognitive function (17). Studies have shown that Tai Chi enhances connections involving the prefrontal cortex, motor cortex, and occipital cortex. Beyond protecting against memory decline, Tai Chi has also been shown to enhance myogenic activity, sympathetic activity, and endothelial cell metabolism (45).

### Co-occurrence Frequency and Centrality of Keywords

The top keywords in the co-occurrence and centrality analyses were “Alzheimer's disease” “older adult,” “Tai Chi,” “quality of life,” and “exercise.”

#### Alzheimer's Disease

Alzheimer's disease is a form of dementia whose most common clinical manifestations are slow onset, gradual loss of memory, and inability to learn new information (46). Alzheimer's disease is characterized by high morbidity and mortality, as well as high healthcare costs. Patients with Alzheimer's disease may lose their ability to live independently until death. Although there is currently no effective treatment (47), early monitoring of cognitive decline and dementia is key to preventing the occurrence and slowing the progression of Alzheimer's disease (47).

#### Older Adults

During normal aging, cognitive function declines to a certain extent. Once the degree of decline exceeds the normal range, the individual may be diagnosed with mild cognitive dysfunction (2). Age is an important risk factor for dementia (46), the prevalence of which also increases with age (37). The incidence of Alzheimer's disease in people over 85 years old is 14 times higher than that in people aged 65–69 years old (48). Given increases in life expectancy and population aging, the individual and public health burden of cognitive decline will continue to increase.

#### Quality of Life

Poor cognitive function reduces quality of life, impairs the ability to live independently, and increases the burden on individuals and society.

#### Exercise

Exercise can effectively prevent age-related cognitive decline and neurodegenerative disease (49). Due to the lack of effective drugs to treat cognitive function, exercise plays an important role in controlling cognitive decline (50). The guidelines for the diagnosis of dementia suggest that patients should adhere to a program of moderate-intensity aerobic exercise to reduce the risk of dementia. At the same time, there is strong evidence that TCHEs (such as Tai Chi and Qigong) can promote the improvement of cognitive function (40).

Dementia is the leading cause of functional disability (51), and several studies have indicated that regular systematic exercise can reduce cognitive symptoms in patients with dementia (52, 53). Research has also demonstrated that physical exercise increases blood flow, promotes hippocampal nerve regeneration, and prevents cognitive decline (54). In addition, it is safe and inexpensive (53). TCHEs are a form of aerobic exercise and mainly include Tai Chi, Qigong, Wuqinxi, and Liuzijue. The practice of TCHEs emphasizes the coordination of breathing and body movements as well as meditation. Conscious control of posture and body movements is necessary during the exercises, helping to stimulate and regulate the nervous system. TCHEs are therefore more comprehensive than other exercise interventions. A previous study reported that Tai Chi training improves connections involving the pre-frontal cortex, motor cortex, and occipital cortex. Such training also increases myogenic activity, strengthens functional connectivity in the brain, improves cognitive function, and protects against memory decline (45). Baduanjin can significantly decrease resting state functional connectivity between the bilateral dorsolateral pre-frontal cortex and the left putamen and insula, thereby leading to improvements in mental control (55). Compared with Tai Chi, few studies have examined the impact of Wuqinxi, Baduanjin, Yijinjing, and other TCHEs on cognitive function. This may be because limited numbers of TCHE-related papers are published in foreign journals. The international influence of Wuqinxi, Baduanjin, Qigong, Yijinjing, and Liuzijue is still weak. Another possible reason is that few studies have investigated the effects of these forms of Wuqinxi, Baduanjin, Yijinjing on cognitive function.

Different types of exercise interventions may have different effects on the effectiveness of the intervention (56). Previous studies have indicated that aerobic exercise is more effective for improving cognitive function than anaerobic exercise (57). In addition, both medium- and high-intensity exercise have been proven to provide multiple health benefits, and even low-intensity exercise is associated with significant health benefits in older adults (58). Some studies suggest that high-intensity interval training (HITT) can induce more significant improvements in cognitive function than moderate-intensity continuous training (59–61). HITT is an emerging training method used in exercise prescription and complementary therapy for the rehabilitation of patients with related chronic diseases (60). But at the same time, HITT can be difficult and may even lead to sports injuries or increase negative emotions in beginners, which is not conducive to long-term practice (62). Since most patients with cognitive impairment are middle-aged or older adults who cannot perform high-intensity exercise, low-to-medium exercise intensity is an adequate alternative for them. Compliance is higher for moderate-intensity exercise than for exercise at other intensities (63). TCHEs are typical representatives of moderate-intensity aerobic exercise with a wide range of adaptability (64), and some studies have shown that this type of exercise is useful for improving cognitive function (20, 65–67).

In terms of the duration of the exercise intervention, even a short intervention time has been associated with significant cognitive benefits. A single episode of moderate-to-vigorous physical activity can improve cognition, and more benefits are obtained as the intervention time increases (58).

TCHEs are not limited by a training venue or training equipment; therefore, they are more convenient and economical for continuous training than other forms of exercise intervention. Furthermore, TCHEs not only improve physical function but also effectively reduce the risk of falls in older adults (63, 68). Research indicates that TCHEs also exert positive effects in patients with type 2 diabetes (69, 70), chronic obstructive pulmonary disease (71, 72), Parkinson's disease (73, 74), and other conditions (75–77).

### Limitations

To our knowledge, the present study is the first bibliometric analysis to evaluate the impact of TCHEs on cognitive function. We analyzed the annual number of publications and citations; the distribution of publications based on countries, and institutions; keywords and references with the strongest citation burst; the status of current research; and potential future trends. Despite the extent of this analysis, our study had some limitations. First, since CiteSpace cannot accurately process data from multiple databases simultaneously and is not available for many databases, the analysis was limited to articles in the WoS database, which may have resulted in a publication bias. Second, to better present the results of the analysis and to ensure the quality of the included literature, only articles and reviews published in English were included. This may have led to a screening bias. Lastly, we did not discuss the distribution of authors in this study due to the limitations of the WoS database, which can only extract the abbreviations of authors' names. This may have decreased the accuracy of the analysis of author-related content.

## Conclusion

The present study involved a bibliometric analysis of research studies related to the use of TCHEs for improving cognitive function conducted from 2001 to 2020. A total of 662 institutions participated in the development of 406 articles, and the number of articles published each year gradually increased. The main research institutions were universities in China and the United States. Most research journals were founded in the United States, mainly in the fields of medicine, sports, nursing, and psychology. *Evidence-based Complementary and Alternative Medicine* was identified as the most productive journal. “Alzheimer's disease,” “exercise,” and “dementia” were the top keywords. Although this study had certain limitations, it constitutes a reference for future research and highlights the potential of TCHEs for managing cognitive decline.

## Data Availability Statement

The original contributions presented in the study are included in the article/supplementary material, further inquiries can be directed to the corresponding author.

## Author Contributions

TF and WL: conceptualization. WL: methodology, writing—original draft preparation, and writing—review and editing. LW: software. TF: investigation, data curation, and supervision. LW and WL: resources. LW and QX: visualization. All authors have read and agreed to the published version of the manuscript.

## Funding

This study was funded by the National Social Science Fund of China (Project Number 17CTY024). This study was also supported by Shanghai University of Sport.

## Conflict of Interest

The authors declare that the research was conducted in the absence of any commercial or financial relationships that could be construed as a potential conflict of interest.

## Publisher's Note

All claims expressed in this article are solely those of the authors and do not necessarily represent those of their affiliated organizations, or those of the publisher, the editors and the reviewers. Any product that may be evaluated in this article, or claim that may be made by its manufacturer, is not guaranteed or endorsed by the publisher.
